# Eggshell pigment composition covaries with phylogeny but not with life history or with nesting ecology traits of British passerines

**DOI:** 10.1002/ece3.1960

**Published:** 2016-02-12

**Authors:** Kaat Brulez, Ivan Mikšík, Christopher R. Cooney, Mark E. Hauber, Paul George Lovell, Golo Maurer, Steven J. Portugal, Douglas Russell, Silas James Reynolds, Phillip Cassey

**Affiliations:** ^1^ Centre for OrnithologySchool of BiosciencesCollege of Life & Environmental SciencesUniversity of BirminghamEdgbastonBirminghamB15 2TTUK; ^2^ Department of Analytical ChemistryFaculty of Chemical TechnologyUniversity of PardubicePardubiceCzech Republic; ^3^ Department of Animal and Plant SciencesUniversity of SheffieldSheffieldS10 2TNUK; ^4^ Department of PsychologyHunter College and the Graduate Center of the City University of New York695 Park AveNew York CityNew York10065; ^5^ Division of Psychology, Social and Health SciencesAbertay UniversityDundeeDD1 1HGUK; ^6^ School of Biological SciencesUniversity of AdelaideAdelaideSouth Australia5005Australia; ^7^ School of Biological SciencesRoyal HollowayUniversity of LondonEghamSurreyTW20 0EXUK; ^8^ Bird GroupDepartment of Life SciencesNatural History MuseumAkeman StreetTringHertfordshireHP23 6APUK

**Keywords:** Biliverdin, eggshell coloration, eggshell pigment, phylogeny, protoporphyrin

## Abstract

No single hypothesis is likely to explain the diversity in eggshell coloration and patterning across birds, suggesting that eggshell appearance is most likely to have evolved to fulfill many nonexclusive functions. By controlling for nonindependent phylogenetic associations between related species, we describe this diversity using museum eggshells of 71 British breeding passerine species to examine how eggshell pigment composition and concentrations vary with phylogeny and with life‐history and nesting ecology traits. Across species, concentrations of biliverdin and protoporphyrin, the two main pigments found in eggshells, were strongly and positively correlated, and both pigments strongly covaried with phylogenetic relatedness. Controlling for phylogeny, cavity‐nesting species laid eggs with lower protoporphyrin concentrations in the shell, while higher biliverdin concentrations were associated with thicker eggshells for species of all nest types. Overall, these relationships between eggshell pigment concentrations and the biology of passerines are similar to those previously found in nonpasserine eggs, and imply that phylogenetic dependence must be considered across the class in further explanations of the functional significance of avian eggshell coloration.

Bird eggs display a great diversity in appearance (i.e., size, shape, background color, and extent of maculation, Fig. 1) across avian lineages (Kennedy and Vevers 1976; Cassey et al. 2012a). Numerous hypotheses have been suggested to explain this functional diversity (summarized in Kilner 2006; Cherry and Gosler 2010; Maurer et al. 2011a), but no single (unifying) hypothesis is likely to explain the striking diversity of eggshell coloration and maculation of extant species; most functional hypotheses are constrained within certain ecological contexts. As an example, eggshell crypsis (Lack 1958) is more likely for ground‐nesting than hole‐nesting species. This strongly suggests that the purported functions of eggshell coloration are not mutually exclusive.

**Figure 1:**
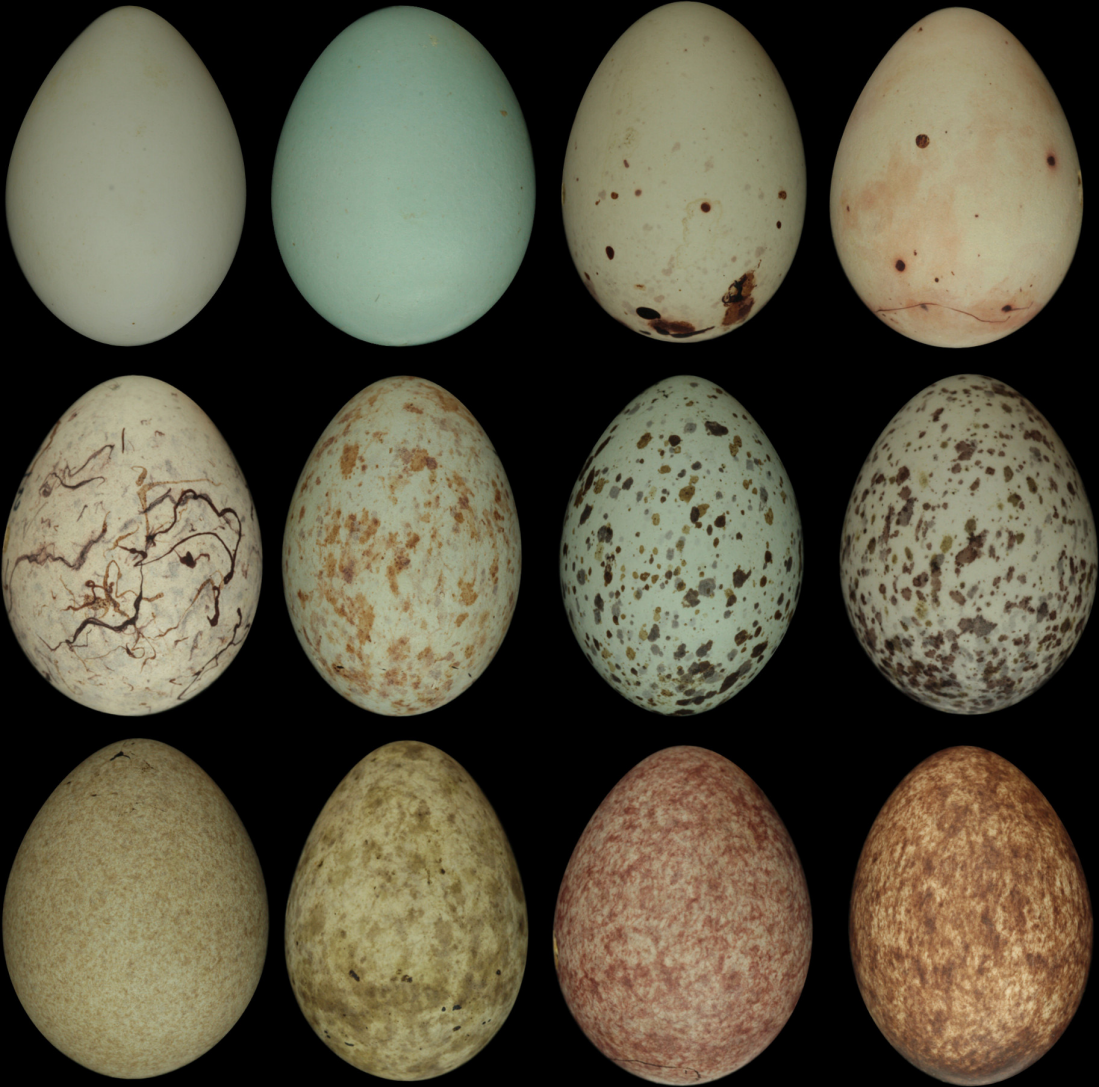
The color and pigment patterns of eggshells vary greatly across avian lineages. We examined eggshells from 71 British breeding passerine species to ascertain the relative importance of nesting ecology, life history, and phylogenetic signature in explaining eggshell pigment concentration and its resultant coloration. From top left to bottom right: dipper (*Cinclus cinclus*), whinchat (*Saxicola rubetra*), common crossbill (*Loxia curvirostra*), chaffinch (*Fringilla coelebs*), Yellowhammer (*Emberiza citrinella*), ring ouzel (*Turdus torquatus*), jackdaw (*Corvus monedula*), wood warbler (*Phylloscopus sibilatrix*), jay (*Garrulus glandarius*), reed warbler (*Acrocephalus scirpaceus*), tree pipit (*Anthus trivialis*), and tree sparrow (*Passer montanus*).

Concentrations of the two principal eggshell pigments (Gorchein et al. 2009), protoporphyrin IX, responsible for red‐brown hues, and biliverdin IX*α*, responsible for blue‐green hues (Kennedy and Vevers 1973, 1976), robustly underpin the coloration of eggshells of nonpasserine species (Cassey et al. 2012a). These pigments have been detected in eggshells of extinct paleognath species (Igic et al. 2010) and in fossils of an oviraptorosaur species (Wiemann et al. 2015), suggesting that these pigments are basal to the avian lineage and have been conserved throughout the diverse radiation of Aves. Passerines comprise about 60% of extant bird species (Sibley and Monroe 1990), and although they display a relatively uniform morphology, they exhibit a wide diversity of behavior and ecology, having colonized a diverse set of ecological niches. For these reasons, passerines provide an interesting context in which to study the role of phylogeny in explaining, and contributing to, the diversity of eggshell pigment concentrations and their resultant coloration.

The nesting ecology and life history of a species are predicted to be important factors in determining eggshell functions, and may therefore largely dictate a species' eggshell color (Kilner 2006). For example, nest humidity, egg microbial content, female body condition, predation risk, and brood parasitism may all influence eggshell coloration (reviewed in Maurer et al. 2011a). The chemical composition (i.e., pigment concentration) and physical (i.e., egg‐shell thickness and the number and tsructure of pores) requirements of the egg and that of the embryo inside may have been central in selection pressures on structural egg traits (Maurer et al. 2011a, 2015). In eggshells of nonpasserines, protoporphyrin concentration was associated with a higher likelihood of cavity‐ and ground‐nesting, whereas biliverdin concentration was associated with a higher likelihood of non‐cavity‐nesting and biparental provisioning (Cassey et al. 2012a). Although eggshell coloration has been extensively studied, to what extent shared phylogenetic signatures, nesting ecology, and life‐history traits may play in determining eggshell pigment concentration in passerines, a group thought to have split off from its sister group 50–55 million years ago (Jarvis et al. 2014), is still largely unknown. The coloration of eggs laid by some species may not be determined by their present‐day ecology at all, but simply be due to their evolutionary history.

Here, we used eggshells from a subset of closely related, but highly ecologically diverse, species across the Passeriformes to ascertain the relative importance of nesting ecology, life history, and phylogenetic signature in explaining eggshell pigment concentrations and their resultant coloration. To assess this diversity, we accessed museum eggshells of 71 British breeding passerine species, across 47 genera and 25 families, which were provided to us on a ‘destructive loan’ (Russell et al. 2010). First, we examined whether there was a phylogenetic basis for variation in eggshell pigment concentrations across species. Then, we examined how eggshell pigment concentrations vary with the nesting ecology and the life history of species while controlling for phylogenetic relationships between the taxa.

## Methods

### Eggshell samples

Eggshells were made available for chemical analyses through a destructive loan of British breeding bird species stored at the Natural History Museum (Bird section), Tring, UK (Russell et al. 2010). These eggs were obtained prior to 1954 by private collectors and lacked sufficient provenance data to be included in the main scientific collection (Russell et al. 2010). For each species, three eggs were chosen at random from different collections to ensure that eggs did not originate from the same clutch, female or location. A full list of species is provided for each of the samples used (Appendix S1). Museum specimens have been used in a number of studies of eggshell coloration (e.g., Cassey et al. 2010a; Portugal et al. 2010; Maurer et al. 2012). Time in storage can affect pigment concentrations and/or the color of eggshells, however, these effects are minimal when eggs are not exposed to artificial light for protracted periods of time and therefore should not have an impact on inter‐species comparisons (Cassey et al. 2010b; Navarro and Lahti 2014).

Data were generated from half‐eggshells only, with the remaining halves being used in a separate study (see Maurer et al. 2012 for further details). Half‐eggshells were cleaned in deionized water and dried at room temperature for 48 h. Eggshell thickness was measured to an accuracy of 1 *μ*m using a modified digital micrometer (series 227‐203, Absolute Digimatic, Mitutoyo, Kawasaki, Japan) at a constant pressure setting of 1.5 N (see Maurer et al. 2011b for further details). Thickness was measured twice at the widest point (equator) of the eggshell.

Eggshells were photographed using a Canon 450D digital camera with a 105 MM Sigma AF lens under standardized conditions following Cassey et al. (2010a). The camera was mounted on a Kaiser camera stand, surrounded by two Calumet photographic umbrellas with silver‐white (AU3046) and flat white (AU3045) linings. Eggs were lit to the right and front using two Osram 11 W energy‐saving light bulbs. Photographs were taken at ISO 400 with an aperture of f16 and an automatic exposure. Eggshells were photographed against a black velvet background. All photographs included a scale bar. The surface area of the half‐eggshell was estimated from photographs using the ‘Egg Area Measurement’ plugin (Troscianko 2014) in ImageJ (Rasband 1997).

### Pigment quantification

Eggshell pigment (i.e., protoporphyrin IX and biliverdin) concentrations were quantified by high‐performance liquid chromatography (HPLC) as previously described in detail by Mikšík et al. (1996) and Cassey et al. (2012a).

### Colorimetry of sample eggshell color

Each image was processed separately. Processing comprised two main phases: selection of the required regions of the image for analysis and calculation of the image statistics. To select the required regions, the image was partitioned into ‘egg’ and ‘background’ using a simple binary threshold on a gray scale version of the image (after Otsu 1979) to locate where the intraclass variance was minimized, and the interclass variance was maximized.

**Figure 2:**
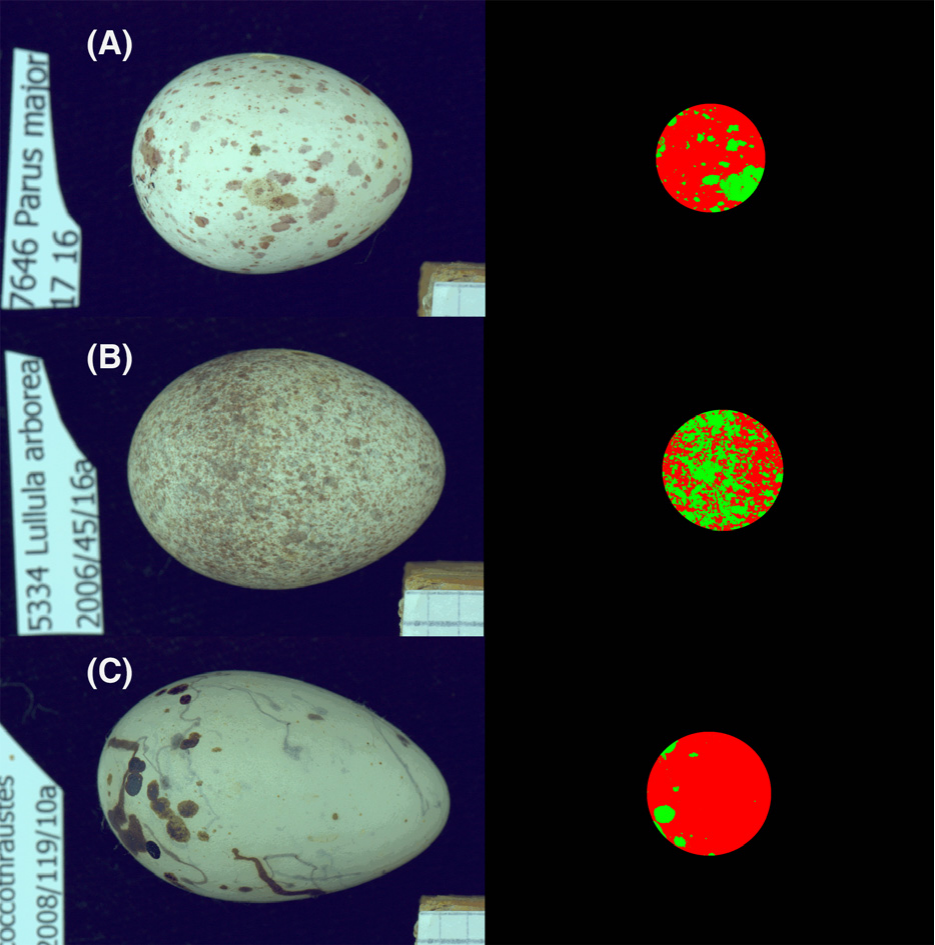
Photographs showing how images were partitioned into egg and background regions, and then how the circular subsample was subsequently identified, for eggs of (A) great tit (*Parus major*), (B) woodlark (*Lullula arborea*), and (C) hawfinch (*Coccothraustes coccothraustes*). (Photos: G. Maurer).

Having defined the ‘egg’ region of the image, a circular subsample comprising a third of the egg area was identified (Fig. 2). The circular subsample was located by taking the centroid (i.e., the mean, midX) of the *x* and *y* pixels of the whole egg area. The centroid was then shifted toward the broadest region of the eggshell using the following equation: midX (after) = midX (before)*0.88. In effect, this approach excluded pixels near the edge of the shell, thereby excluding parts of the image where spot shapes and brightness levels may have been distorted by curvature.

All digital images were in the standardized RAW format. Images were converted from linearized RGB to human XYZ (see Lovell et al. 2005) and consequently to CIELAB color space using the MATLAB image processing toolbox (The Mathworks, Natick, MA, 2000). In the CIELAB color space, the L* channel corresponds to saturation of the color, the a* channel corresponds to red (+)/green (−) color values, and the b* channel corresponds to yellow (+)/blue (−) values.

Pixels were categorized as being either maculated or nonmaculated using a kmeans clustering algorithm (*k *=* *2, giving a target of two centroids), allowing the degree of maculation (i.e., percentage of dark pixels) present in the subsample to be calculated. For the nonmaculated pixels (i.e., background), we calculated the mean and standard deviation of the L*a*b* color for all pixels (see Lovell et al. 2013 for more details).

### Comparative life‐history and nesting ecology traits

Life‐history data were collected from Birds of the Western Palaearctic (Cramp and Simmons 1978–1994), Handbook of the Birds of the World (Del Hoyo et al. 1992–2011), and from family‐ and species‐specific monographs (for more details see Cassey et al. 2012a). Life‐history and nesting ecology traits of species were defined as: clutch size – the mean number of eggs laid per breeding attempt; adult body mass (g) – mean body mass of adults (males and females); parasitized – hosts to brood parasites; nest type – open or cavity‐nesting (i.e., tree, burrow); and nest location – ground or off ground (i.e., shrub, tree).

### Phylogenetic tests

Phylogenetic relationships between passerine species were constructed using the phylogeny of breeding British birds, constructed using molecular data from 249 species (Thomas 2008). To measure the strength of the phylogenetic signal in protoporphyrin and biliverdin pigment concentrations, we estimated Pagel's lambda (*λ*) (Pagel 1999; Freckleton et al. 2002), using the package MOTMOT (Thomas & Freckleton 2012) in R version 3.1.1 (R Development Core Team 2011). Pagel's *λ* varies between 0, phylogenetic independence, and 1, a trait which covaries in direct proportion to a species' shared evolutionary history, consistent with a Brownian‐motion model of trait evolution (Freckleton et al. 2002).

### Statistical analysis

**Figure 3:**
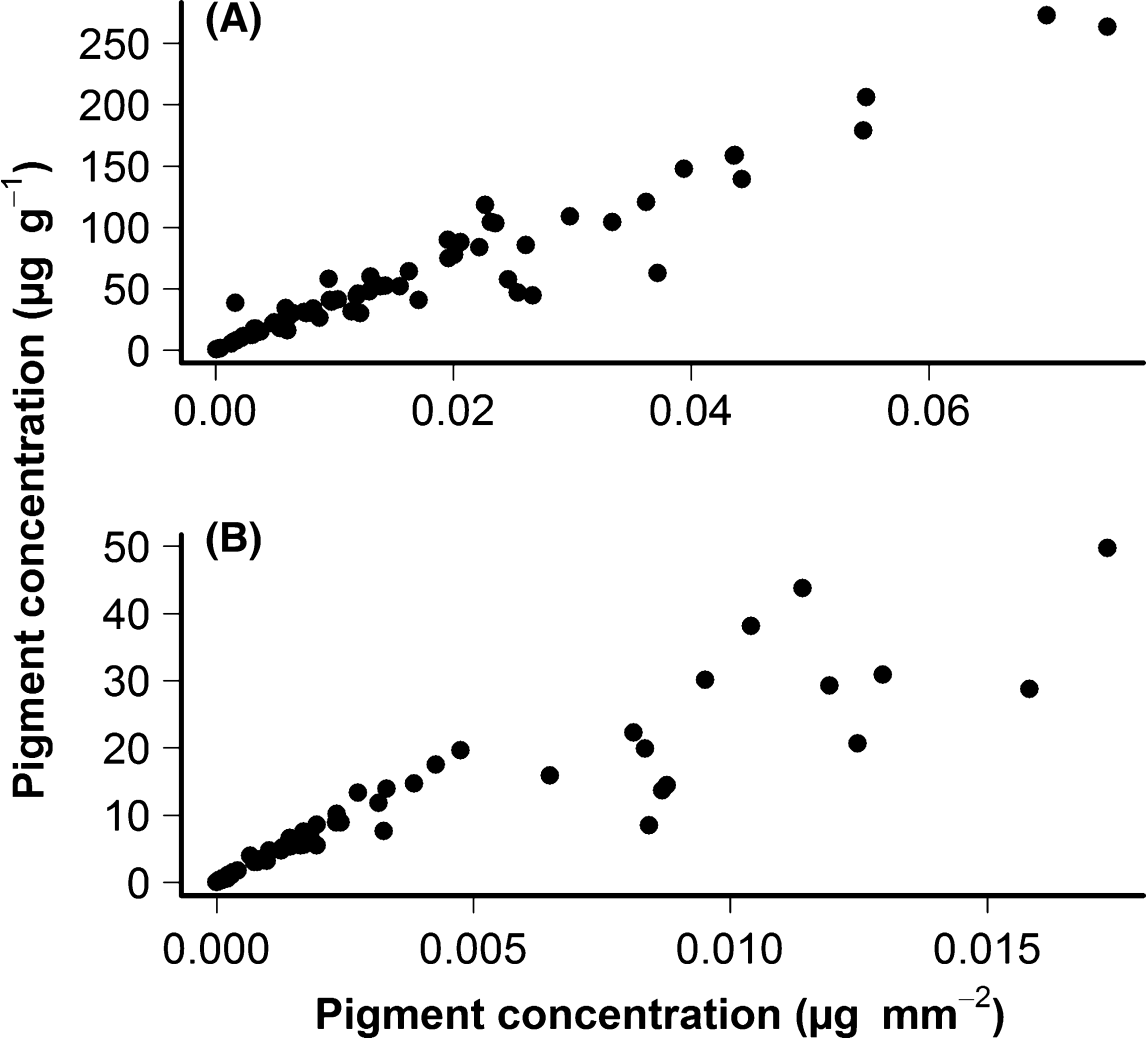
Scatterplot of the relationship between the mean concentrations standardized by mass (*μ*g g^−1^) and surface area (*μ*g mm^−2^) of eggshells for (A) protoporphyrin and (B) biliverdin in eggshells of 71 British passerine species.

All statistical analyses were performed in R version 3.1.1 (R Development Core Team 2011). The mean sample pigment concentrations per mass of eggshell (*μ*g g^−1^) were, across species, strongly and positively correlated to those per surface area (*μ*g mm^−2^) for both protoporphyrin (Spearman's correlation, *ρ* = 0.97, *n* = 71, *P *<* *0.0001; Fig. 3A) and biliverdin (*ρ* = 0.94, *n* = 71, *P *<* *0.0001; Fig. 3B). For the remaining analysis, the eggshell pigment concentrations were standardized by eggshell mass only.

Statistical models examining the influence of life‐history and nesting ecology traits on eggshell pigment concentrations (*μ*g g^−1^ eggshell) were constructed controlling for the nonindependence of species relationships through shared common ancestry using phylogenetic generalized linear models (pgls) in the package ‘caper’ (Orme et al. 2012). Full models used the following traits as explanatory variables: eggshell thickness (*μ*m), clutch size, nest type, nest location, adult body mass (g), and host to brood parasites. Mean values per species were used.

Models were ranked using Akaike's information criterion (AIC) scores and weights (Akaike 1974). Model‐averaged estimates were calculated using a subset of models in which the cumulative sum of the AIC model weights was >0.95 using the package ‘MuMIn’ (Barton 2010). Traits were deemed as having an important influence on the model if the 95% confidence interval (CI) around the model‐averaged mean effect estimate did not include zero.

## Results

### Pigment concentration and eggshell appearance

Eggshell pigment concentrations (*μ*g g^−1^) were associated with variation in eggshell colorimetrics (Table 1). Higher protoporphyrin concentrations were associated with lower saturations (L*) of background color, higher red (+a*) background color content, and higher degrees of eggshell maculation (Table 1). Higher biliverdin concentrations were associated with lower saturations (L*) of background color and higher green (−a*) background color content (Table 1).

**Table 1:** Model‐averaged mean effect estimates and 95% confidence intervals (CIs) testing the influence the concentrations of two eggshell pigments, protoporphyrin IX and biliverdin IX*α* (log_10_ *μ*g g^−1^), on eggshell colorimetrics using eggs of 71 species of British passerines. Models were constructed controlling for the nonindependence of species relationships through shared common ancestry using phylogenetic generalized linear models (see Methods for more details)

Eggshell colorimetrics	Protoporphyrin (*μ*g g^−1^ eggshell)			Biliverdin (*μ*g g^−1^ eggshell)		
	Model effect estimate (95% CI)	Relative variable importance	*N* containing models	Model effect estimate (95% CI)	Relative variable importance	*N* containing models
Intercept	3.54 (2.80 to 4.28)			2.79 (1.85 to 3.73)		
a*	**0.03 (0.0009 to 0.05)**	0.59	1	**−0.06 (−0.09 to −0.03)**	1.00	3
L*	**−0.03 (−0.05 to −0.02)**	1.00	3	**−0.04 (−0.06 to −0.03)**	1.00	3
Maculation (%)	**0.01 (0.008 to 0.02)**	1.00	3	0.003 (−0.004 to 0.01)	0.25	1
b*	0.011 (−0.004 to 0.03)	0.20	1	−0.01 (−0.03 to 0.02)	0.20	1

Significant estimates using 95% CIs are labeled in bold.

### Phylogenetic patterns in eggshell coloration

**Figure 4:**
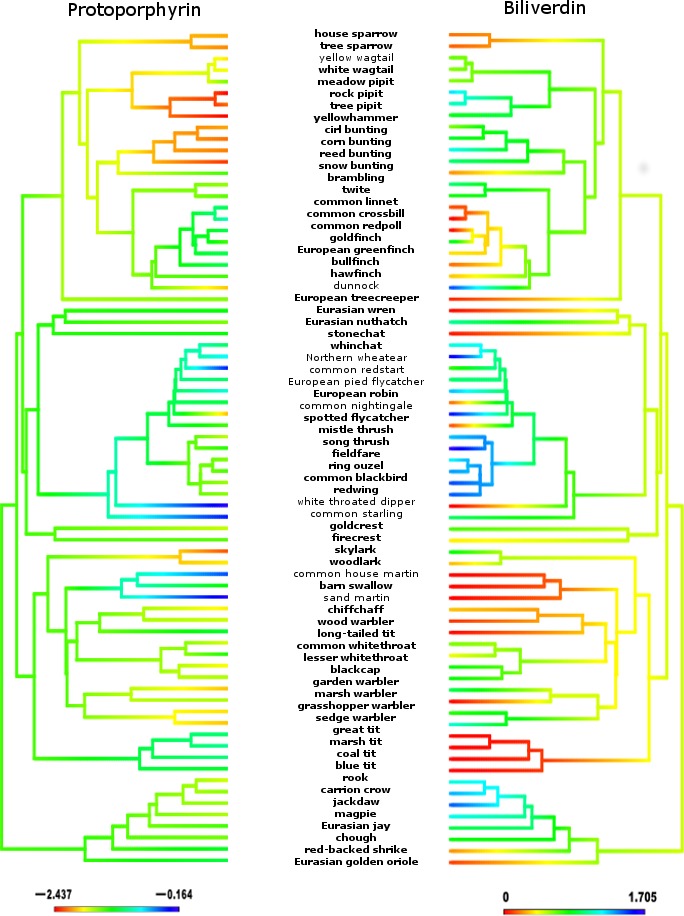
Phylogenetic tree for 71 British passerine species used in a comparative analysis investigating the relationship between eggshell pigment concentrations and species' breeding biology. The colored branches (i.e., the trait value) illustrate the concentration (log_10_) of the two pigments protoporphyrin IX (left) and biliverdin (right). Species laying maculated eggshells are labeled in bold.

Replicate measures of eggshell pigment concentration (within species) were more repeatable (intraclass correlation coefficients [5th and 95th percentiles]) for protoporphyrin (0.76 [0.67 and 0.83, respectively]) than for biliverdin (0.62 [0.50 and 0.73, respectively]). Both pigments are highly conserved phylogenetically (protoporphyrin = 1.00 [0.902, 1.00]; biliverdin = 1.00 [0.879, 1.00], Fig. 4), signifying that phylogenetic relatedness is of paramount importance when considering pigment concentrations and coloration of species' eggshells in multispecies comparisons.

### Comparative life‐history and nesting ecology traits

Eggshell protoporphyrin and biliverdin concentrations (standardized by eggshell mass only) were positively related (Table 2). Cavity‐nesting species laid eggs with lower protoporphyrin concentrations in the shell, while higher biliverdin concentrations were associated with thicker eggshells for species of all nest types (Table 2).

**Table 2:** Model‐averaged mean effect estimates and 95% confidence intervals (CIs) testing the influence of the concentrations of two eggshell pigments, protoporphyrin IX and biliverdin IX*α* concentrations (log_10_ *μ*g g^−1^), on life‐history and nesting ecology traits using eggs of 71 species of British passerines. Models were constructed controlling for the nonindependence of species relationships through shared common ancestry using phylogenetic generalized linear models (see Methods for more details)

Life‐history and nesting ecology traits	Protoporphyrin (*μ*g g^−1^ eggshell)			Biliverdin (*μ*g g^−1^ eggshell)		
	Model effect estimate (95% CIs)	Relative variable importance	*N* containing models	Model effect estimate (95% CIs)	Relative variable importance	*N* containing models
Intercept	1.65 (0.62, 2.69)			−0.71 (−1.70, 0.29)		
Biliverdin concentration (*μ*g g^−1^ eggshell)	**0.36 (0.16, 0.57)**	1.00	47	n/a	n/a	n/a
Protoporphyrin concentration (*μ*g g^−1^ eggshell)	n/a	n/a	n/a	**0.43 (0.21, 0.66)**	1.00	50
Nest location (ground/off ground)	0.002 (−0.18, 0.18)	0.20	19	−0.15 (−0.37, 0.07)	0.46	24
Parasitized (yes/no)	0.11 (−0.08, 0.30)	0.35	19	−0.11 (−0.31, 0.10)	0.33	22
Nest type (cavity/open)	0.19* (−0.03, 0.40)	0.60	27	0.03 (−0.23, 0.28)	0.21	18
Adult body mass	−0.28 (−0.88, 0.33)	0.34	21	0.28 (−0.39, 0.95)	0.39	24
Clutch size	−0.09 (−0.18, 0.003)	0.70	29	0.06 (−0.04, 0.15)	0.36	23
Eggshell thickness	2.29 (−7.91, 12.49)	0.27	20	6.47* (−0.64, 13.58)	0.61	26

Significant estimates using 95% CIs are labeled in bold, and estimates which are significant using 90% CIs are followed by an ‘*’.

## Discussion

We have shown that eggshell pigment concentration, and hence their resultant coloration, of 71 British passerine species is largely explained by a species' phylogeny and to a lesser extent by its nesting ecology and life‐history traits.

As predicted, protoporphyrin concentration was positively related to the red background coloration of the eggshell and increased maculation. Protoporphyrin has reflectance peaks at 441 and 557.2 nm, which correspond to the brown‐red region of the spectrum (Poole 1965). Biliverdin concentration was related to green background coloration of the eggshell. Biliverdin has absorbance peaks at 375 and 665 nm, which correspond to the blue‐green region of the spectrum (Falchuk et al. 2002), and has previously been related to blue and green eggshell coloration (e.g., Cassey et al. 2012a). Despite these aforementioned relationships, the variability detected in the associations between eggshell pigment concentrations and visible color (to the human eye) is considerable and reinforces previous research demonstrating that visible coloration alone cannot be used to determine the presence/absence of eggshell pigments (Cassey et al. 2012a,b; Brulez et al. 2014).

Birds have tetrachromatic color vision and are especially sensitive to UV light (reviewed in Bennett and Cuthill 1994; Cuthill 2006). The colors which eggshell pigments emit occupy only a small component (~0.10%) of the avian color space (Cassey et al. 2012a,b; Hanley et al. 2015). This constraint in chromatic variation in eggshells, in relation to total color vision of birds, suggests that we need to reconsider the importance of visual functions of eggshell pigments. The perceived color of an eggshell is not solely dependent on its pigment concentration but also on the spectral properties of the ambient light and reflectance spectra (Endler 1990). Nanostructural mechanisms, for example, the calcium carbonate matrix (Hanley et al. 2015), are also important as the structure of the eggshell cuticle can enhance UV reflectance (Fecheyr‐Lippens et al. 2015) and even produce glossiness and iridescence in eggs laid by some species [e.g., great tinamou, *Tinamus major* (Igic et al. 2015)].

Eggshell protoporphyrin and biliverdin concentrations were positively related (Table 2), supporting the proposition that the two pigments are most likely derived from the same precursor metabolic pathway (Wang et al. 2009). Eggshell concentrations standardized by shell mass were, across species, strongly and positively correlated to those standardized by shell surface area, suggesting that pigments occurring in the outermost layer are representative of pigments spread throughout the eggshell. However, for eggshells containing higher concentrations of biliverdin, there is an indication that for a given value of eggshell mass, there is more pigment in the surface of the eggshell than there is spread throughout the shell (Fig. 3A).

Pigment concentrations of eggshells exhibited strong covarying phylogenetic patterns (Fig. 4), concurring with results obtained from a similar destructive analysis of eggshells of British nonpasserine species (Cassey et al. 2012a). Results using 95% CIs as the basis for parameter estimation did not find any significant correlations between eggshell pigment concentrations and a species' nesting ecology and life‐history traits; however, when the CIs were lowered to 90% (Table 2), some parameters reached significance. These traits may still be important in determining a species' eggshell pigment concentrations but display high variability among species and would potentially require an analysis of eggshells from a greater number of species to reach statistical significance.

Open‐nesting species laid eggs containing higher concentrations of protoporphyrin in their eggshells (90% CIs; Table 2), which translates into redder eggs with increased maculation. There are two main strategies that birds employ to make their eggs more cryptic (reviewed in Stoddard et al. 2011): background matching (Wallace 1889; Stevens and Merilaita 2009a) where eggs match the nesting environment, and disruptive coloration (Cott 1940; Stevens and Merilaita 2009b) where markings on eggs make it more difficult for the receiver (e.g., predator) to distinguish the true outline of the egg, thereby creating confusion in discerning egg shapes in the nest. Protoporphyrin‐related background coloration (i.e., red/brown hues) and maculation can enable eggs to be more cryptic using both of these strategies. In contrast, for those species nesting in cavities, eggshell color might be driven by alternative strategies. Species laying eggs containing lower concentrations of pigment could promote greater light transmission through the eggshell, facilitating embryonic development (Maurer et al. 2015), or allow eggs to be more visible to parents under low‐light conditions.

We found that species laying eggs with thicker shells contained higher concentrations of biliverdin in their eggshells (90% CIs; Table 2). Both increased eggshell thickness and increased pigment concentration reduce the amount of light filtered through to the developing embryo (Maurer et al. 2015). Eggs of open‐nesting species (i.e., those species laying eggs containing higher concentrations of biliverdin) are more exposed to light and require mechanisms to limit the amount of harmful UV light to pass through to the embryo. The light transmitted inside the eggshell corresponds to light reflected from the surface, especially in the blue‐green spectrum (Maurer et al. 2015), which acts as a stimulant of the embryonic circadian rhythm (Csernus et al. 1999). Therefore, by laying eggshells containing biliverdin, species ensure that the light which does pass through is beneficial to the embryo.

In conclusion, shell pigment concentrations, and their resultant coloration of eggs laid by passerines, are largely explained by the evolutionary history of species in a multispecies comparison and only to a lesser extent by nesting ecology and life‐history traits. These results, combined with similar results found for eggs of 49 species of nonpasserines, indicate that a species' evolutionary history may be much more important in determining a species' eggshell pigment concentrations and their resultant color than previously thought. We strongly encourage future studies to consider the phylogenetic signature of a species because the functional significance of eggshell coloration may be intractable without appropriate consideration of its evolutionary history.

## Conflict of Interest

The authors declare no competing financial interests.

## Supporting information


**Appendix S1.** Species list. Scientific nomenclature follows www.birdtree.org (Jetz W., G. H. Thomas, J. B. Joy, K. Hartmann, and A. O. Mooers (2012). The global diversity of birds in space and time. Nature 491:444−448).
