# Supplementary material for: Eggshell pigment composition covaries with phylogeny but not with life history or with nesting ecology traits of British passerines
Source: Ecol Evol. 2016 Feb 12;6(6):1637–45. doi: 10.1002/ece3.1960 (PMC4752363; doi:10.1002/ece3.1960)
Supplement: Supplementary file 1 — Appendix S1. Species list. Scientific nomenclature follows www.birdtree.org (Jetz W., G. H. Thomas, J. B. Joy, K. Hartmann, and A. O. Mooers (2012). The global diversity of birds in space and time. Nature 491:444−448). [file ECE3-6-1637-s001.docx]

**Appendix S1:** Species list. Scientific nomenclature follows www.birdtree.org (Jetz W., G. H. Thomas, J. B. Joy, K. Hartmann, and A. O. Mooers (2012). The global diversity of birds in space and time.

Nature 491:444−448).

**Species (scientific name) Species (common name)**

| *Acrocephalus palustris* | Marsh warbler |
| --- | --- |
| *Acrocephalus schoenobaenus* | Sedge warbler |
| *Acrocephalus scirpaceus* | (Eurasian) Reed warbler |
| *Aegithalos caudatus* | Long-tailed tit |
| *Alauda arvensis* | (Eurasian) Skylark |
| *Anthus petrosus* | Rock pipit |
| *Anthus pratensis* | Meadow pipit |
| *Anthus trivialis* | Tree pipit |
| *Linaria cannabina* | (Eurasian) Linnet |
| *Carduelis carduelis* | (European) Goldfinch |
| *Carduelis chloris* | (European) Greenfinch |
| *Carduelis flavirostris* | Twite |
| *Certhia familiaris* | (Eurasian) Treecreeper |
| *Cinclus cinclus* | (white-throated) Dipper |
| *Coccothraustes coccothraustes* | Hawfinch |
| *Corvus frugilegus* | Rook |
| *Corvus monedula* | (Eurasian) Jackdaw |
| *Delichon urbicum* | (Northern) House martin |
| *Emberiza cirlus* | Cirl bunting |
| *Emberiza citrinella* | Yellowhammer |
| *Emberiza schoeniclus* | Reed bunting |
| *Erithacus rubecula* | (European) Robin |
| *Ficedula hypoleuca* | (European) Pied flycatcher |
| *Fringilla coelebs* | (Eurasian) Chaffinch |
| *Fringilla montifringilla* | Brambling |
| *Garrulus glandarius* | (Eurasian) Jay |
| *Hirundo rustica* | Barn swallow |
| *Lanius collurio* | Red-backed shrike |
| *Locustella naevia* | (common) Grasshopper warbler |
| *Loxia curvirostra* | Red crossbill |
| *Lullula arborea* | Woodlark |
| *Luscinia megarhynchos* | (common) Nightingale |
| *Miliaria calandra* | Corn bunting |
| *Motacilla alba* | White wagtail |
| *Motacilla cinerea* | Grey wagtail |
| *Motacilla flava* | Yellow wagtail |
| *Muscicapa striata* | Spotted flycatcher |
| *Oenanthe oenanthe* | (Northern) Wheatear |
| *Oriolus oriolus* | (Eurasian) Golden oriole |
| *Parus ater* | Coal tit |
| *Parus caeruleus* | Blue tit |
| *Parus major* | Great tit |
| *Parus palustris* | Marsh tit |
| *Passer domesticus* | House sparrow |
| *Passer montanus* | (Eurasian) Tree sparrow |
| *Phoenicurus phoenicurus* | (common) Redstart |
| *Phylloscopus collybita* | (common) Chiffchaff |
| *Phylloscopus sibilatrix* | Wood warbler |
| *Pica pica* | (black-billed) Magpie |
| *Plectrophenax nivalis* | Snow bunting |
| *Prunella modularis* | Hedge Accentor |
| *Pyrrhocorax pyrrhocorax* | (red-billed) Chough |
| *Pyrrhula pyrrhula* | (Eurasian) Bullfinch |
| *Regulus ignicapilla* | Firecrest |
| *Regulus regulus* | Goldcrest |
| *Riparia riparia* | Sand martin |
| *Saxicola rubetra* | Whinchat |
| *Saxicola torquatus* | (common) Stonechat |
| *Sitta europaea* | (wood) Nuthatch |
| *Sturnus vulgaris* | (common) Starling |
| *Sylvia atricapilla* | Blackcap |
| *Sylvia borin* | Garden warbler |
| *Sylvia communis* | (common) Whitethroat |
| *Sylvia curruca* | Lesser whitethroat |
| *Troglodytes troglodytes* | (winter) Wren |
| *Turdus iliacus* | Redwing |
| *Turdus merula* | (Eurasian) Blackbird |
| *Turdus philomelos* | Song thrush |
| *Turdus pilaris* | Fieldfare |
| *Turdus torquatus* | ring ouzel |
| *Turdus viscivorus* | Mistle thrush |
